# Antimicrobial resistance before and after the COVID-19 pandemic in Northern Brazil

**DOI:** 10.1017/ash.2026.10341

**Published:** 2026-04-13

**Authors:** José Eudes de Carvalho Neri, Julius Caesar Mendes Soares Monteiro, Lorena Luciane Martins Rodrigues, Edgar de Brito Sobrinho, Cristiane Guimarães Monte, Ana Carolina Paes Boulhosa, Rafaela Caroline Amador Ferreira, Rita Catarina Medeiros Sousa

**Affiliations:** 1 Tropical Diseases Graduate Program, Belém, Pará, 66055-240, Brazil; 2 Federal University of Pará, Belém, Pará, 66075-110, Brazil; 3 Brazilian Association of Intensive Care Medicine, São Paulo, São Paulo, 04545-100, Brazil; 4 Brazilian Company of Hospital Services (EBSERH), Belém, Pará, 66073-000, Brazil

## Abstract

**Objective:**

This study evaluates the impact of the COVID-19 pandemic on the profile of bacterial resistance in healthcare-associated lower respiratory tract infections (HAI/LRTI), in an intensive care unit.

**Setting:**

City of Belém-PA.

**Design:**

This is a retrospective and analytical cross-sectional study, in which the resistance profile of HAI/LRTI bacterial isolates was evaluated over 2018–2022.

**Results:**

The review of HAI notifications revealed 330 lower respiratory tract infections during the study period. The bacteria with a significant change in the resistance profile between the period pre-COVID and the post-COVID periods were *P. aeruginosa* (*P* = .011), *K. pneumoniae* (*P* < .001) and *A. baumannii* (*P* = .001), with increased profiles multidrug-resistant, and extensively drug-resistant, and strains with pandrug-resistant profile, in 2020 and 2021. In the analysis by antibiotic class, there was a significant increase in *A. baumannii* resistance to carbapenems and *K. pneumoniae* resistance to carbapenems.

**Conclusions:**

Comparing the periods, there was an emergence of *K. pneumoniae* resistant to aminoglycosides and carbapenems; of *P. aeruginosa* with tendency to resistance to aminoglycoside, carbapenem, 4th generation cephalosporin and anti-pseudomonal penicillin + beta-lactamase inhibitor; of *A. baumannii* resistant to aminoglycoside, carbapenem, quinolone, anti-pseudomonal penicillin + beta-lactamase inhibitor and penicillin + beta-lactamase inhibitor and 4th generation cephalosporin.

## Introduction

The outbreak of severe acute respiratory syndrome coronavirus 2 (SARS-CoV-2) infection was initially reported in December 2019, in Wuhan, China. The virus rapidly spread worldwide, affecting approximately 775 million individuals by January 2024 and causing 7,019,704 deaths in the same period, according to the WHO data.^1^ Among the clinical manifestations reported, the pulmonary form has emerged as the predominant factor necessitating hospitalization, with beds being consistently occupied in wards, intensive care units (ICUs), and semi-ICUs for long periods and the possibility of bacterial co-infection, the indiscriminate use of antimicrobials had substantially increased during this period.^2,3^

Multidrug-resistant (MDR) bacteria in hospital-acquired infections (HAI) significantly influence the patients’ clinical response, treatment cost, and the occurrence of hospital outbreaks. The WHO reported that the annual death rate directly caused by MDR bacteria is predicted to increase to approximately 10 million by 2050.^4^

In 2017, the WHO published a list of priority antimicrobial-resistant pathogens that require the research and development of new antibiotics. The list comprised 12 families of bacteria that threaten human life, causing bacteremia, sepsis, and events that result in high morbidity and mortality rates.^5^ The WHO list was divided into three categories according to the urgency of need for new antibiotics: critical, high, and medium priority. The causative agents of HAIs were classified as follows: CRAB, CR-PA, CRE as critical priority pathogens, and VRE and MRSA as high priority pathogens. Other agents were also listed, with greater emphasis on those causing community infections.^6,7^

Hospitalized patients are at high risk of acquiring an HAI. Healthcare facilities can serve as sources of outbreaks or exacerbate pathogen transmission, potentially leading to community-wide spread of infections. Statistics indicate that out of every 100 hospitalized patients, 7 will contract an HAI, with the risk doubling and increasing up to 20 times higher in underdeveloped and developing countries.^8^ The mortality rates increase by two to threefold when infections are resistant to antimicrobials. In addition, the collective experience accumulated over the past 2 years amidst the COVID-19 pandemic unequivocally demonstrates that both patients and healthcare workers face a heightened risk of SARS-CoV-2 infection during the provision of healthcare services, emphasizing the imperative need for protective measures.^9^

We aimed to assess the impact of the coronavirus disease 2019 (COVID-19) pandemic on the bacterial resistance profile by examining the reports of LRT infections in the ICU of a private hospital located in the city of Belém, Pará.

## Materials and methods

### Study design

This cross-sectional, retrospective, and analytical study was conducted from 2018 to 2022 in the state of Pará. Data obtained through a review of HAI notifications from patients admitted to an ICU were used in this study.

### Definition of healthcare-associated infections

Healthcare-associated infections were defined according to standardized surveillance criteria applied by the Hospital Infection Control Committee, based on national and international guidelines incorporating clinical, radiological, and microbiological parameters for pneumonia, ventilator-associated pneumonia, tracheobronchitis, and ventilator-associated tracheobronchitis.^7,9^

Events were classified as hospital-acquired when infection onset occurred ≥48 hours after hospital admission and met standardized surveillance criteria for healthcare-associated lower respiratory tract infections. Definitions for pneumonia, ventilator-associated pneumonia, tracheobronchitis, and ventilator-associated tracheobronchitis followed national infection surveillance guidelines issued by the Brazilian Health Regulatory Agency, aligned with international infection prevention and control recommendations. Case classification incorporated objective clinical, radiological, and microbiological parameters rather than subjective clinical judgment.

### Characterization of the study population

The study included records of HAIs of the lower respiratory tract (pneumonia, ventilator-associated pneumonia, tracheobronchitis, and ventilator-associated tracheobronchitis), notified by the Hospital Infection Control Commission of the participating hospital, from January 2018 to December 2022.

### Study site

The study hospital is a private, not-for-profit, tertiary care institution that only serves users of supplementary health insurance. It is located in Belém, in the state of Pará, in the north of Brazil, and currently has 170 active beds. Initially, the ICU comprised 30 beds. In response to the onset of the COVID-19 pandemic, additional 35 beds were made available during the peak months. Currently, the ICU is equipped with 37 beds.

### Inclusion criteria

We included adult patients (≥18 years) with ICU stays longer than 48 hours who developed their first case of healthcare-associated lower respiratory tract infection, confirmed by microbiological culture.

### Exclusion criteria

The exclusion criteria were individuals readmitted to a hospital within 6 months after discharge, cultures with incomplete sensitivity profile results and patients transferred from other healthcare institutions.

### Data consolidation, presentation, and analysis

After data collection, the infections were divided into two periods: period 1 (P1) (from January 2018 to March 2020) and period 2 (P2) (from April 2020 to December 2022), before and after the first confirmed case of COVID-19 in the city.

Antimicrobial susceptibility testing followed BrCAST-EUCAST standards.^10,11^Results were categorized as susceptible, intermediate (susceptible with increased exposure), or resistant. Resistance profiles were classified as MDR, extensively drug-resistant (XDR), and pandrug-resistant (PDR) according to international consensus definitions.

The primary outcome was the comparison of overall antimicrobial resistance profiles (MDR/XDR/PDR) between the pre and postpandemic periods. Analyses by antibiotic class were considered secondary exploratory outcomes. Given the exploratory nature of these analyses, no formal correction for multiple comparisons was applied, and results were interpreted cautiously with emphasis on consistent resistance trends.

The data were analyzed using descriptive and inferential statistics. Pearson’s χ^2^ test, G-test, and Fisher’s exact test were used to evaluate the association between the independent and dependent variables. The statistical program SPSS (Statistical Package for the Social Sciences) version 20.0 was used for data tabulation and analysis, with the significance level set at 5% for all tests.

The clinical cutoff point tables were derived from the BrCAST-EUCAST manual, which has been standardized at the institution since 2018.

### Ethical considerations

The study was submitted to the Research Ethics Committee of the Tropical Medicine Center of the Federal University of Pará, in accordance with the CNS/MS resolution 466/12, governing research involving human beings, a process that began in November 2021 (BRAZIL, 2012) (approved under CAAE: 57818621.5.0000.5172 on June 9, 2022).

## Results

### General analysis of HAI/LRTI

**Figure 1.**
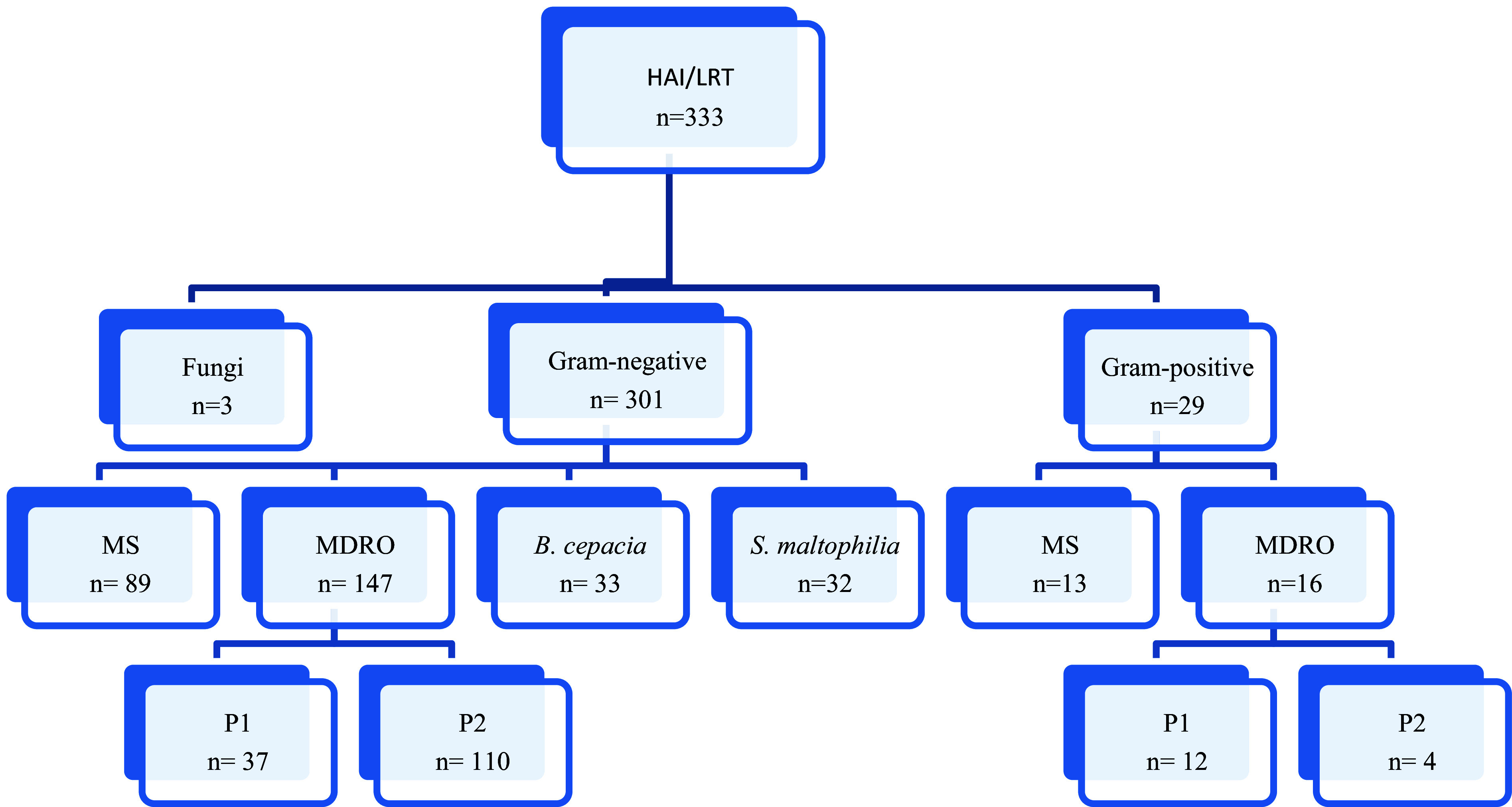
Distribution of HAI/LRTI isolates, by sensitivity profile, between the years 2018 and 2022, distinguishing the bacteria that cannot be classified in relation to the resistance profile, S. maltophilia and B. cepacia.HAI: Hospital-acquired infections, LRTI: Lower respiratory tract, MS: Multisensitive, MDRO: Multidrug-resistant microorganism. *Source:* HICC, 2023.

Based on the HAI reports for the years 2018 to 2022, drawn up by the HICC of the hospital studied, 333 patients who developed HAIs in the lower respiratory tract identified through microbiological isolation were reported in the ICUs. Among these patients, three acquired infections caused by fungi. Hence, only 330 patients with bacterial isolates were included in the study after exclusion of fungal infections (n = 3) (Figure 1).

The patient’s age ranged from 21 to 96 years (mean: 73.26 ± 16.55 years (median 77)). In P1, the prevalence of infections was higher in patients aged ≥60 years (n = 96, 86.4%). In P2, the patient’s age ranged from 19 to 96 years (mean: 70.61 ± 16.18 years (median 73)). Similar to P1, the prevalence remained higher in patients aged ≥60 years in P2 (n = 175, 78.8%).

In terms of gender, HAI occurred in 64 (57.8%) male patients in P1 and 130 (58.4%) male patients in P2.

**Figure 2.**
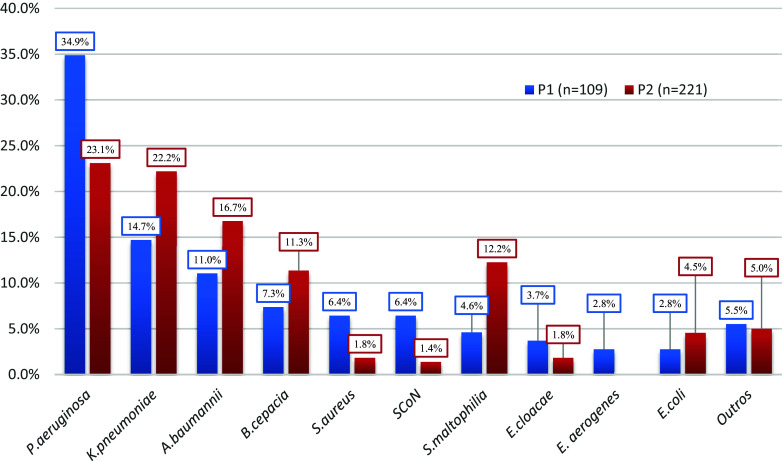
Distribution of microorganisms isolated from a patient with Healthcare-Associated Infection of the lower respiratory tract, in the period P1 (before the start of the COVID-19 pandemic) and P2 (after the start of the COVID-19 pandemic). *Source:* HICC, 2023.

The distribution of bacterial isolates is shown in Figure 2, in the pre and postpandemic periods.

### HAI/LRTI samples and sites

In terms of clinical specimen, most positive cultures in P1 were derived from tracheal secretions (n = 74, 67.9%), followed by blood samples (n = 32, 29.4%) and bronchoalveolar lavage (n = 3, 2.8%). Similarly, in the P2 samples, the predominant sources of positive cultures were tracheal secretion (n = 166, 75.1%), blood samples (n = 52, 23.5%), and bronchoalveolar lavage (n = 3, 1.4%). Positive cultures from tracheal aspirates were considered indicative of infection only when associated with compatible clinical signs, radiological findings, and standardized surveillance criteria for healthcare-associated lower respiratory tract infections applied by the Hospital Infection Control Committee, and were not interpreted in isolation to distinguish colonization from infection. Blood cultures were attributed to a respiratory source when bacteremia occurred in the presence of concurrent clinical and radiological evidence of lower respiratory tract infection and in the absence of an alternative infectious focus. All bronchoalveolar lavage samples met institutional adequacy criteria for specimen quality and were processed according to standardized microbiological protocols.

VAP accounted for 32.1% (n = 35) of infections detected in P1, and it accounted for more than 60% (n = 136) of infections reported in P2, followed by tracheobronchitis associated with mechanical ventilation, 18.6% (n = 41).

### Analysis of the antimicrobial resistance of *Klebsiella pneumoniae*

The isolated *K. pneumoniae* strains were significantly more resistant to amikacin, ertapenem, gentamicin, imipenem and meropenem (Table 1).

**Table 1.** Comparison of antibiotic resistance rates of *K. pneumoniae* bacterial isolates, in periods P1 and P2. S: sensitive, R: resistant

Antibiotic	P1		P2		*P* value	OR
	S	R	S	R		
Amikacin	11 (68.8)	5 (31.3)	17 (34.7)	32 (65.3)	.0220	4.14 (IC 95% 1.21–12.52)
Gentamicin	13 (81.3)	3 (18.8)	19 (38.8)	30 (61.2)	.0467	3.47 (IC 95% 1.03–10.40)
Ceftriaxone	4 (25)	12 (75)	3 (7.9)	35 (92.1)	.1771	3.88 (IC 95% .90–16.70)
Cefepime	4 (25)	12 (75)	6 (12.2)	43 (87.8)	.2453	2.38 (IC 95% .66–8.53)
Piperacillin/Tazobactam	7 (43.8)	9 (56.3)	10 (20.4)	39 (79.6)	.0998	3.03 (IC 95% .91–9.6)
Ertapenem	11 (68.8)	5 (31.3)	16 (34.8)	30 (65.2)	.0227	4.12 (IC 95% 1.19–12.66)
Imipenem	16 (68.8)	5 (31.3)	17 (34.7)	32 (65.3)	.0018	6.02 (IC 95% 1.7–17.03)
Meropenem	12 (75)	4 (25)	18 (36.7)	31 (63.3)	.0101	5.16 (IC 95% 1.44–15.96)
Ciprofloxacin	6 (37.5)	10 (62.5)	8 (17.4)	38 (82,6)	.1619	2.85 (IC 95% .82–10.17)
Levofloxacin	6 (40)	9 (60)	2 (25)	6 (75)	.6674	1.80 (IC 95% .31–10.75)
Tigecycline	13 (86.7)	2 (13.3)	5 (100)	0	>.9999	.00 (IC 95% .00–6.66)
Sulfamethoxazole/Trimethoprim	8 (53.3)	7 (46.7)	21 (44.7)	26 (55.3)	.7671	1.41 (IC 95% .48–4.39)
Polymyxin B	3 (50)	3 (50)	16 (53,3)	14 (46,7)	>.9999	.87 (IC 95% .18–4.24)

Source: HICC, 2023.

### Analysis of the antimicrobial resistance of *Pseudomonas aeruginosa*

The rate of *P. aeruginosa* resistance to carbapenems, quinolones, and fourth-generation cephalosporins was already high before the COVID-19 pandemic (Table 2).

**Table 2.** Comparison of antibiotic resistance rates of *P. aeruginosa* bacterial isolates, in periods P1 and P2. S: sensitive, R: resistant

Antibiotic	P1		P2		*P* value	OR
	S	R	S	R		
Amikacin	34 (89.5)	4 (10.5)	42 (82.4)	9 (17.6)	.3837	1.82 (IC 95% .49–5.68)
Gentamicin	31 (81.6)	7 (18.4)	7 (58.3)	5 (41.7)	.1292	3.16 (IC 95% .77–11.66)
Cefepime	17 (44.7)	21 (55.3)	20 (40)	30 (60)	.6696	1.21 (IC 95% .51–2.87)
Piperacillin/Tazobactam	24 (63.2)	14 (36.8)	25 (49)	26 (51)	.2034	1.78 (IC 95% .77–4.04)
Imipenem	20 (54.1)	17 (45.9)	25 (49)	26 (51)	.6712	1.22 (IC 95% .52–2.89)
Meropenem	25 (65.8)	13 (34.2)	26 (51)	25 (49)	.1967	1.84 (IC 95% .78–4.19)
Ciprofloxacin	22 (59.5)	15 (40.5)	24 (48)	26 (52)	.3853	1.58 (IC 95% .67–3.91)
Levofloxacin	6 (54.5)	5 (45.5)	5 (62.5)	3 (37.5)	>.9999	.72 (IC 95% .13–4.14)
Polymyxin B	27 (96.4)	1 (3.6)	25 (100)	0	>.9999	–

Source: HICC, 2023.

After the pandemic, no statistical difference was observed in the rates of *P. aeruginosa* resistance to most antibiotic classes.

### Analysis of the antimicrobial resistance of *Acinetobacter baumannii*

In P1, the resistance of the A. baumannii strains exceeded 50% in almost all classes of antibiotics, except polymyxin B. In P2, the aforementioned strains showed complete resistance to six antibiotics, while maintaining a sensitivity rate of 97.1% to polymyxin B (Table 3).

**Table 3.** Comparison of antibiotic resistance rates of *A. baumannii* bacterial isolates, in periods P1 and P2. S: sensitive, R: resistant

Antibiotic	P1		P2		*P* value	OR
	S	R	S	R		
Amikacin	3 (37.5)	5 (62.5)	3 (8.1)	34 (91.9)	.0594	6.8 (IC 95% 1.22–33.77)
Gentamicin	11 (45.5)	6 (54.5)	0	34 (100)	.0049	–
Ampicilina/Sulbactam	5 (41.7)	7 (58.3)	0	10 (100)	.0902	–
Piperacillin/Tazobactam	2 (28.6)	5 (71.4)	0	11 (100)	.1373	–
Cefepime	4 (33.3)	8 (66.7)	0	13 (100)	.0391	–
Imipenem	4 (33.3)	8 (66.7)	0	37 (100)	.0023	–
Meropenem	2 (25)	6 (75)	0	37 (100)	.0283	–
Polymyxin B	8 (88.9)	1 (11.1)	34 (97.1)	1 (2.9)	.3710	.23 (IC 95% .012–4.99
Ciprofloxacin	4 (33.3)	8 (66.7)	1 (2.7)	36 (97.3)	.0100	18 (IC 95% 2.20–224.1)

Source: HICC, 2023.

Screening for carbapenemase genes (NDM, VIM, OXA-48, and KPC) yielded no positive results among the isolates tested.

## Discussion

Among the study population, 57.8% male patients were identified in the prepandemic period, while 58.4% male patients were identified in the postpandemic. Additionally, the age group over 60 years old predominated in both periods. This observation aligns with the report of the Bahçe *et al.’s* study, which compared the profiles of bacterial isolates from tracheal aspirates between the prepandemic (April/2019 to March/2020) and postpandemic (April/2020 to March/2021) periods in Turkey.^12^ Their findings also demonstrated a predominance of male patients in both periods, with an average age range similar to that reported in our study and with no statistical difference between the periods (*P* = .710). Male patients predominated in both periods, reflecting the demographic profile of the ICU population.

Among the microorganisms analyzed, Gram-negative bacteria were the most frequently isolated, accounting for 301 isolates, which constituted 90.3% of the total samples (including Gram positive, Gram negative, and fungi). *P. aeruginosa*, *K. pneumoniae*, and *A. baumannii* were prevalent in both periods. These same GNB were isolated and identified in other studies, but with different frequencies.^12–15^ They have been recognized as the primary agents of lower respiratory tract HAIs in ICU patients even before the COVID-19 pandemic.^16^

Bacterial isolates classified as MDR, XDR, or (PDR) accounted for 45% of HAI/LRTI caused by bacteria in P1 and 51.6% in P2. A single-center study conducted in Italy reported a higher proportion of postpandemic resistance, with 64.5% of MDRO isolates identified in patients with ventilator-associated pneumonia, albeit based on a smaller sample of 48 patients with infections.^3^ In a study of more than 8,000 healthcare-related infections in Brazil, 29.7% were caused by MDROs. One of the possible explanations for this difference between the studies is the number of participants and the locations of the studies. Hence, the local and socioeconomic reality of the countries most affected by the increase in MDR bacteria must be considered.^17^

Several factors have contributed to this scenario. Initial reports from China indicated that over 50% of deaths among COVID-19 patients were associated with secondary bacterial infection.^18^ Kanungo *et al* reported antibiotic prescription in 60%–70% of patients with COVID-19.^19^ Conversely, Rawson *et al*’s (2020) systematic review identified only 8% of bacterial or fungal co-infection in patients with COVID-19, out of 800 individuals.^20^ However, 72% received empirical antibiotic therapy. In India, a multicenter study involving 10 hospitals found that 47% of patients received carbapenems.^21^ During this time, the WHO warned against the indiscriminate use of azithromycin and hydroxychloroquine, emphasizing that there was no recommendation for their use outside of controlled clinical trials.^22^ Shockingly, data indicate that in some areas, 48.53% of patients used antibiotics without medical advice.^23^ Detailed individual antibiotic exposure data and stratified analyses by COVID-19 status were not available for all patients, representing a limitation of this retrospective surveillance-based study.

Other factors may explain the increased prevalence of MDROs: the high intensity of care required by critically ill patients during the COVID-19 pandemic; prolonged patient contact during procedures such as pronation, requiring four to five healthcare professionals (physiotherapist, nursing technician, nurse, and doctor); and the presence of inexperienced ICU professionals.^24^ Most MDRO bacterial infections have developed in critically ill patients, resulting in prolonged hospitalization.^20,25–28^

The widespread use of antibiotics prophylactically or at an early stage has also contributed to bacterial resistance worldwide, with more than half of patients with COVID-19 receiving antibiotics.^25,29,30^ This impact is particularly significant in underdeveloped or developing countries, where infection control programs and the rational use of antimicrobials have not yet been implemented.^31^

The excessive use of antibiotics during the COVID-19 pandemic can be attributed to several factors: similarity of the picture of SARS-CoV-2 infection with community-acquired pneumonia (cough, fever, and radiological infiltrate); anxiety and uncertainty regarding the severity of the disease and its potential complications; the lack of effective medications; and the possibility of co-infection or secondary infection as a justification for administering antibiotics to patients with worsening clinical conditions.^32^ Features such as bilateral ground-glass opacities on imaging, lymphopenia, and the absence of focal lobar consolidation are more characteristic of COVID-19 pneumonia compared with typical community-acquired bacterial pneumonia.

*K. pneumoniae* has emerged as a significant pathogen in healthcare-related infections in the postpandemic period of COVID-19.^15,23,29,33^ Resistance is primarily mediated by the production of β-lactamases and other antibiotic-inactivating enzymes.^34^ In this study, this species showed the highest level of resistance, with isolates classified as resistant to all antibiotic classes tested (PDR), a finding consistent with a study in Saudi Arabia.^33^

In the *P. aeruginosa* isolates, the highest frequency of bacteria causing HAI/LTRI was quantified in the two study periods, with a significant increase in bacteria classified as MDR and XDR. Data from the United States indicated a 32% increase in MDR *P. aeruginosa* during the first peak of the COVID-19 pandemic.^35^ The rate of detection of resistance genes is low in this species, given the predominance of other resistance mechanisms, but with a tendency toward an increase in bla _NDM._^36^ Despite the absence of carbapenemases in the postpandemic period, resistance rates to carbapenems, quinolones, and fourth-generation cephalosporins remained close to over 50%, consistent with findings reported by Bahçe *et al.* ^12^

*A. baumannii* isolates showed an increase in their resistance profile between the periods, with a significant impact on antimicrobial resistance to six antibiotics. These findings align with those of Bazaid *et al*, who found resistance to all the tested antibiotic classes in 65 ICU patients with infections involving different anatomic sites.^33^ Notably, their study revealed that 100% *A. baumannii* isolates showed resistance to carbapenems, 87% to amikacin, and 53% to colistin. Although specific carbapenemase profiles were not identified in these bacteria, other studies showed that _blaOXA-23_, _blaOXA-143_, and _blaOXA-58_ were the most prevalent in *A. baumannii.* ^36,37^

Infection control programs have had major barriers to maintaining or implementing the necessary measures to tackle MDR bacteria during the COVID-19 pandemic.^38^ Gram-negative bacteria are the main agents of healthcare-related infections, with an impact on treatment, contact precautions, and cause of outbreaks in hospital units, and with an increase in quantity and resistance profile in the postpandemic period of COVID-19.^39^ Carbapenemases have increased the resistance potential of these bacteria and are spreading in various regions of the country.^36^ The impact of resistance goes beyond the ICU setting; long-stay institutions have also experienced a shift in their bacterial resistance profile.^40^

Healthcare facilities must develop strategies to address this scenario of MDR bacteria in LRTIs and implement quality of care measures such as mechanical ventilation bundles, initiatives to reinforce the correct technique and adherence to hand hygiene, and proper cleaning of units and devices, which consequently has an impact on healthcare-related infections caused by MDROs.^41^

To combat antimicrobial resistance effectively, laboratory and hospital monitoring needs to be expanded and strengthened, as it is through the data obtained that assertive actions can be drawn up at local, state, and federal levels. Flows, protocols, and trainings in the detection of these resistant bacteria are extremely important to improve the recovery of samples, the investigation of outbreaks, and the notification of critical priority microorganisms in both public and private institutions. Accurate differentiation between colonization and true infection is central to antimicrobial stewardship, as inappropriate treatment of colonization can unnecessarily increase selective pressure for antimicrobial resistance.

The change in the profile of antibiotic resistance directly affects the chances of a cure and the quality of hospital care, given the increase in serious cases, especially respiratory infections, in ICUs.

## Conclusion

Healthcare-associated lower respiratory tract infections in this ICU were predominantly caused by *Pseudomonas aeruginosa, Klebsiella pneumoniae,* and *Acinetobacter baumannii.*

Following the COVID-19 pandemic, a substantial increase in antimicrobial resistance was observed, particularly to carbapenems and aminoglycosides.

These findings underscore the urgent need for strengthened antimicrobial stewardship, robust infection control programs, and continuous resistance surveillance in critical care settings.
